# Telemedicine for Tobacco Cessation and Prevention to Combat COVID-19 Morbidity and Mortality in Rural Areas

**DOI:** 10.3389/fpubh.2020.598905

**Published:** 2021-01-18

**Authors:** Ashley L. Merianos, Bradley Fevrier, E. Melinda Mahabee-Gittens

**Affiliations:** ^1^School of Human Services, University of Cincinnati, Cincinnati, OH, United States; ^2^Department of Public and Allied Health, Bowling Green State University, Bowling Green, OH, United States; ^3^Division of Emergency Medicine, Cincinnati Children's Hospital Medical Center, Cincinnati, OH, United States; ^4^College of Medicine, University of Cincinnati, Cincinnati, OH, United States

**Keywords:** smoking, electronic nicotine delivery systems, smoking cessation, telemedicine, coronavirus disease (COVID)-19

## Introduction

The coronavirus disease 2019 (COVID-19) outbreak has reached pandemic proportions with a large global footprint (1). In December 2019, COVID-19 was first reported in Wuhan, Hubei Province, China (1). On 28 January 2020, the National Health Commission of People's Republic of China released national strategies for assisting prevention efforts for rural areas and the older adult population (2). Nevertheless, this outbreak was not contained, and as of 16 December 2020, it has spread to 191 countries resulting in over 74 million cases and 1,646,687 deaths worldwide (3).

To date, over 16.9 million cases and 307,064 deaths have been reported in the United States (U.S.), with numbers climbing disproportionately on a day-to-day basis (3). New epidemiologic and clinical evidence is constantly evolving related to this infection while the pandemic is hastily progressing worldwide. Rapid efforts are needed to protect vulnerable populations including those living in rural areas. For example, before this pandemic, U.S. urban hospitals were over twice as likely to have growth in number of intensive care unit (ICU) beds available over a 1-year period compared to rural hospitals (4). Thus, rural hospitals are not as likely to have the higher level of resources and medical care needed for this crisis. Preventive and resource allocation strategies [e.g., ventilator availability (5)] are needed to reach and care for vulnerable rural populations.

To help address this public health challenge, telemedicine efforts have greatly expanded to provide a “safety-net” of medical and preventive care for vulnerable populations worldwide (6, 7). Although offering telemedicine services in rural areas is encouraged to reduce in-person infection risks, geographical disparities exist in the access and availability of telemedicine. This is in part due to unreliable internet in some U.S. and international rural areas (7, 8). Economic-related concerns for offering virtual services in rural areas include weakened healthcare system infrastructures, physician shortages, and a disproportionate number of residents having low-income, no health insurance, or inadequate insurance coverage for telemedicine visits (9).

There is a call to action to address COVID-19 disparities that are disproportionately affecting vulnerable communities, including those: living in remote areas, with chronic diseases and risk factors such as tobacco use, and from low socioeconomic backgrounds (10). An early systematic literature review (11) hypothesized that smoking behavior may be associated with adverse progression of COVID-19, and a subsequent meta-analysis discovered that smokers were nearly two times more likely to have severity progression than non-smokers (12). However, there are limited data available on the clinical characteristics of COVID-19 infected individuals living in rural areas. The purpose of this commentary is to review information on existing U.S. tobacco use disparities in rural areas and knowledge available on tobacco use and COVID-19 outcomes. We also provide suggestions on future directions for tobacco cessation efforts reaching rural smokers during this pandemic.

## Tobacco Use and COVID-19

### Tobacco Use and COVID-19 Disparities Among U.S. Rural Adults

Tobacco users living in U.S. rural areas may represent an additional subgroup at risk for higher COVID-19-related morbidity. Irrespective of COVID-19, individuals living in rural areas have high rates of tobacco use and related morbidity compared to urban or metropolitan area dwellers due to several factors including: low income, low educational attainment, higher social acceptability to tobacco use, permissive tobacco control policies, and lack of healthcare access (13, 14). Rural communities are heterogenous in nature, but low socioeconomic indicators largely contribute to the rural-urban disparity gap of poor health behaviors such as smoking and COVID-19 mortality rates (15).

Table 1 shows that U.S. rural residents generally have higher tobacco use rates and lower smoking cessation rates than urban residents (16–19). Rural adult residents also have a high prevalence of smoking-related, long-term health consequences and causes of mortality (20). Over one-quarter (26%) of rural adult residents have one chronic health condition (e.g., diabetes) and about 36% have two or more conditions (21). The top leading causes of death among rural dwellers include cancer, heart disease, chronic lower respiratory disease, and stroke (22). The Centers for Disease Control and Prevention indicates that those deemed high risk for severe COVID-19 are older adults, individuals with medical conditions including those with lung disease, heart conditions, and diabetes, and individuals living in rural communities (23). A meta-analysis (24) suggests smoking and chronic health conditions are the most common underlying factors reported among patients hospitalized for COVID-19. Given that rural residents have high rates of combustible and non-combustible tobacco/nicotine product use and related chronic health conditions that place them at risk for poor health outcomes and excess mortality, it is important to synthesize the evidence available on adverse COVID-19 specific outcomes in this population.

**Table 1:** Prevalence of current tobacco product use overall, and by rural-urban geographical location among U.S. Adults >18 years old.

**Current product use type^a^**	**Overall**	**Rural**	**Urban**
Any tobacco product	25.4%	35.0%	31.0%
Cigarettes	20.6%	28.5%	25.1%
Cigars	4.8%	2.6%	4.6%
Smokeless Tobacco	3.4%	8.6%	6.0%
E-Cigarettes^b^	-	2.8%	2.1%

*e-cigarettes, electronic cigarettes*.

a *Data from SAMHSA, 2017 (16)*.

b *Data from Mumford et al., 2019 (17). Overall e-cigarette prevalence statistic was not provided*.

### Tobacco Use and COVID-19-Related Morbidity and Mortality

The initial epidemiological and clinical data available retrospectively assessed adult patients who sought treatment in Wuhan where COVID-19 first emerged (25–28). It is important to note that approximately 45% of individuals in China reside in rural areas and face similar issues to the U.S. rural population, such as lack of healthcare access and affordability (29, 30). These studies report that patients who presented with COVID-19 had symptoms similar to commonly reported symptoms of tobacco use (e.g., cough, respiratory-related symptoms) as outlined by the U.S. Surgeon General (20). Patients presenting with COVID-19 also had high rates of comorbidities (25–27, 31) that have been causally linked to tobacco use including hypertension, cardiovascular disease, and diabetes (20).

Recent literature reviews found that tobacco use is associated with poor disease progression (32), and that severe COVID-19 outcomes among tobacco users are further perpetuated among those with these pre-existing conditions (33). One study found patients with a smoking history were over 14 times more likely to experience disease progression than patients with no smoking history after adjustment for important factors including patient age (27). Additional research reports that over one-fourth (26%) of current smokers and about 8% of former smokers were admitted to the ICU, needed mechanical ventilation, or died (17). Concerning COVID-19 mortality-related risk factors, of 11 admitted patients who were current smokers in China, five did not survive (28). A more recent U.S. study found that current smokers, when compared to never smokers, were at increased risk of needing mechanical ventilation, having severe-to-critical illness, lower survival time in days, and more deaths (34). Concerning non-combustible tobacco products, a study found that 13–24-year-olds who engaged in exclusive e-cigarette use and dual-use of e-cigarettes and combustible cigarettes were at significantly increased odds to have positive COVID-19 diagnoses, while accounting for clustering by U.S. states and regions (35). Despite limitations of the rapidly emerging available data, such as relying on electronic medical record data for smoking status, it is likely that combustible and non-combustible tobacco product use influences COVID-19 progression and negative health outcomes including death. Thus, a recent review concluded that smoking cessation is expected to reduce COVID-19-related risks and severe complications, and acknowledged telemedicine as one tool that can be employed to promote quitting smoking (36).

### Telemedicine for the Treatment of Tobacco Use and Dependence During and Beyond COVID-19

A recent epidemiologic model that has informed related policy suggests that public health measures including physical distancing would be needed for 3 months to decrease the COVID-19 peak impact on the healthcare system (37). The model suggests these measures should be maintained intermittently over 12–18 months while the disease remains detected in individuals and until a vaccine is made widely available (37). Social distancing mandates naturally reinforce the need to rapidly implement or augment communication modalities that can be used with individuals and groups who may not be able to meet face-to-face and live in multiple locations (38). Fortunately, there are many tobacco cessation programs that have already expanded their reach to rural tobacco users with high prevalence rates (39) who may have limited access to in-person programs, irrespective of COVID-19 restrictions. These programs have demonstrated acceptability and efficacy and include smokefree.gov and other internet-based programs (40, 41), telephone programs (e.g., tobacco Quitline) (42) and mobile phone-based programs (e.g., texting) (43). Therefore, it is important to expand the reach of telemedicine to address the rural-urban geographic inequities and improve the access, delivery, and efficiency of preventive health services among rural areas globally (7).

### Promoting Evidence-Based Treatment for Tobacco Use and Dependence Delivered via Telemedicine to Rural Areas During and Beyond COVID-19

In light of the concerns that tobacco use increases the risk of COVID-19-related morbidity and mortality, measures are needed to mitigate illness progression in infected individuals. Given concerns about contracting COVID-19 and evidence of possible increased risk for smokers and vapers, this pandemic may have a positive impact on decreasing smoking and vaping rates. During this pandemic, telemedicine modalities can continue to provide, expand, and improve the delivery of evidence-based information, which includes the “5 A's of tobacco cessation (44).” The first step is to Ask about tobacco use—this would be complete if the smoker has enrolled into a program. The second step is to Advise to quit—during this step, telemedicine-based programs should capitalize on this “teachable moment” (45) by incorporating messaging that leverages concerns about COVID-19 and the benefits of quitting smoking and/or vaping as a way to reinforce the “Advise” portion. There are increases in smoking abstinence rates when cessation interventions are conducted during “teachable moments” such as during children's visits for acute illnesses (46), during cancer screenings (47), when having a cancer diagnosis (48), when initiating HIV treatment (49), and during pregnancy (50). The third step is to Assess readiness to quit—this can be easily assessed by phone-, video-, or web-based programs. The fourth step is to Assist in quitting—this includes the recommendation to offer pharmacotherapy such as nicotine replacement therapy, which has shown efficacy in increasing cessation rates (51). The fifth step is to Arrange follow-up—during this time of social isolation, tobacco users need help and support. The knowledge that there are many options such as online chat groups or telephone counselors that are available will provide them with much needed support and encouragement.

Incorporating telemedicine during this critical period may increase its use among remote and hard-to-reach rural populations over time. One U.S. cancer center successfully used telemedicine to deliver individual and group tobacco treatment virtually to tobacco-dependent cancer patients (52). They reported improved attendance and engagement at telehealth visits compared to prior in-person visits. Another study conducted among current smokers <25 years old who participated in the Youth Quitline services in Hong Kong found that over 4-in-10 agreed the pandemic was a motivator to quit tobacco use, over 7-in-10 changed their tobacco use habits due to public health measures (e.g., wearing a mask), and nearly 6-in-10 reduced their daily cigarette consumption (53). Therefore, telemedicine has the potential to decrease patient-level barriers to in-person tobacco cessation treatment among all ages worldwide, such as lack of transportation, childcare, and travel costs. Moreover, the opportunity to offer continuous cessation encouragement and support may also increase quit rates during and beyond COVID-19 restrictions.

## Discussion

Public health campaigns that reach vulnerable rural populations are highly warranted to promote the availability of remote tobacco cessation and prevention initiatives during this pandemic. Rural populations have higher rates of poverty, chronic illnesses, poor healthcare access, and lack of insurance—all of which are risk factors for COVID-19 (8, 9). Thus, they could greatly benefit from low-cost telemedicine programs that are easily accessible to decrease tobacco-related morbidity. Some countries have implemented tobacco product sales bans and other policy measures during the pandemic to help mitigate possible viral transmission (e.g., sharing tobacco products), which provides an exceptional opportunity to decrease the global tobacco use burden (54). In addition to telemedicine services, these measures include leveraging new technology to encourage and support tobacco users to quit smoking with evidence-based resources. For example, the tobacco Quitline number could be promoted among rural smokers and vapers. Healthcare professionals and tobacco treatment specialists who interact with rural residents, for example, should be involved in offering evidence-based tobacco cessation interventions via telemedicine. Large-scale public health interventions delivered via telemedicine should be targeted to tobacco users who live in rural areas.

## Author Contributions

AM conceptualized the article, drafted the manuscript, and approved the final manuscript as submitted. BF conceptualized the article, drafted the manuscript, and approved the final manuscript as submitted. EM-G conceptualized the article, drafted the manuscript, and approved the final manuscript as submitted. All authors contributed to the article and approved the submitted version.

## Conflict of Interest

The authors declare that the research was conducted in the absence of any commercial or financial relationships that could be construed as a potential conflict of interest.
